# Combinatorial Effects of Transcutaneous Spinal Stimulation and Task-Specific Training to Enhance Hand Motor Output after Paralysis

**DOI:** 10.46292/sci23-00040S

**Published:** 2023-11-17

**Authors:** Jeonghoon Oh, Michelle S. Scheffler, Erin E. Mahan, Shane T. King, Catherine A. Martin, Jenny Dinh, Alexander G. Steele, Marcia K. O'Malley, Dimitry G. Sayenko

**Affiliations:** 1 Department of Neurosurgery, Center for Translational Neural Prosthetics and Interfaces, Center for Neuroregeneration, Houston Methodist Research Institute, Houston, Texas, USA;; 2 Department of Mechanical Engineering, Rice University, Houston, Texas, USA

**Keywords:** hand motor function, spinal cord injury, spinal cord stimulation, stroke

## Abstract

**Background:**

Despite the positive results in upper limb (UL) motor recovery after using electrical neuromodulation in individuals after cervical spinal cord injury (SCI) or stroke, there has been limited exploration of potential benefits of combining task-specific hand grip training with transcutaneous electrical spinal stimulation (TSS) for individuals with UL paralysis.

**Objectives:**

This study investigates the combinatorial effects of task-specific hand grip training and noninvasive TSS to enhance hand motor output after paralysis.

**Methods:**

Four participants with cervical SCI classified as AIS A and B and two participants with cerebral stroke were recruited in this study. The effects of cervical TSS without grip training and during training with sham stimulation were contrasted with hand grip training with TSS. TSS was applied at midline over cervical spinal cord. During hand grip training, 5 to 10 seconds of voluntary contraction were repeated at a submaximum strength for approximately 10 minutes, three days per week for 4 weeks. Signals from hand grip dynamometer along with the electromyography (EMG) activity from UL muscles were recorded and displayed as visual feedback.

**Results:**

Our case study series demonstrated that combined task-specific hand grip training and cervical TSS targeting the motor pools of distal muscles in the UL resulted in significant improvements in maximum hand grip strength. However, TSS alone or hand grip training alone showed limited effectiveness in improving grip strength.

**Conclusion:**

Task-specific hand grip training combined with TSS can result in restoration of hand motor function in paralyzed upper limbs in individuals with cervical SCI and stroke.

## Introduction

While the underlying causes and mechanisms of cervical spinal cord injury (SCI) and stroke are different, each can contribute to substantial upper limb (UL) paralysis that often results in a significant loss of independence in an individual’s daily life.^1^,^2^ It is critical, therefore, to systematically examine methods that have the potential to promote functional recovery of the UL. Specifically, a combinatorial approach of rehabilitation and neuromodulation strategies aimed at targeting recovery of specific UL function following SCI and stroke represent critical elements in the management of the UL paralysis, as they have the potential to promote functional recovery and enhance quality of life.

Although communication between the brain and spinal cord may be disrupted, modulation of the excitability of intact sublesional spinal circuits could enhance their responsiveness to descending inputs through the remaining cortico-spinal pathways.^3^,^4^ Noninvasive, transcutaneous electrical spinal stimulation (TSS) is an emerging electrical neuromodulation strategy that has the potential to increase the excitability of spinal circuits and facilitate functional recovery after paralysis. Recent studies demonstrated that cervical TSS after SCI, either alone or in combination with physical training, facilitated the recovery of UL functions.^5^-^7^ Additionally, epidural spinal cord stimulation (ESS) has been previously introduced as a novel approach to activate neural networks and enable a variety of functions after SCI.^8^-^10^ In a recent study, ESS resulted in notable enhancements in grip strength and hand motor function in individuals poststroke.^11^ Despite the positive results in UL motor recovery following both TSS and ESS in individuals after cervical SCI or stroke, there has been limited exploration of TSS as a targeted approach for hand motor recovery poststroke in particular. In addition, the potential benefits of combining hand grip training with TSS for individuals with UL paralysis after cervical SCI have not been thoroughly investigated. Specifically, previous studies have investigated the effectiveness of cervical TSS^5^,^12^ or ESS^13^ in improving UL function, but it is yet to be determined whether task specific grip strength training is necessary or if any training with TSS/ESS will be sufficient to improve grip function. The mechanisms underlying improved UL function due to electrical neuromodulation require further investigation to clarify these questions and inform the development of effective rehabilitation strategies for individuals with cervical SCI.

Therefore, the purpose of our case series is to investigate the combinatorial effects of task-specific training and cervical TSS to restore hand motor deficits in both individuals with cervical SCI and stroke. We hypothesized that participants who underwent the combined training approach of grip training plus TSS (GripTr+TSS) would demonstrate enhanced volitional hand function compared to those who received either hand grip training alone (GripTr+Sham) or TSS alone (NoTr+TSS) in individuals after stroke and after cervical SCI.

## Methods

### Participants

**Table 1** presents the demographic information of the study participants. Four participants with cervical SCI classified as American Spinal Cord Injury Association Impairment Scale (AIS) A and B and two participants with cerebral stroke were enrolled in the study. Participants with SCI had a chronic injury (1 year) and were classified using the International Standards for Neurological Classification of Spinal Cord Injury (ISNCSCI) examination as having a neurological level of injury at or between C4-C7. The two participants with stroke had experienced either ischemic or hemorrhagic stroke, as shown in **Table 1**. All participants gave informed consent to experimental procedures, which were approved by the local ethics committee. eTable 1 presents the ISNCSCI upper limb motor scores for individuals with cervical spinal cord injury and the Fugl-Meyer Assessment of Upper Extremity Function (FMAUE) motor scores for those with stroke across the different sessions. One of the participants (SCA-022) was unable to complete the phase involving GripTr+Sham due to personal reasons.

**Table 1.** Demographics of study participants

	**ID**	**Age, years**	**Gender**	**AIS**	**NLI**	**Postinjury, years**	**Trained hand**
**SCI**	SCA-014	25	Male	A	C4	14	Right
	SCB-019	29	Male	B	C4	7	Left
	SCC-021	27	Male	B	C6	2	Left
	SCA-022	23	Male	A	C4	4	Left
	**ID**	**Age, years**	**Gender**		**Type**	**Postinjury, years**	**Side affected by stroke/Trained hand**
**Stroke**	STL-007	59	Female	Ischemic		2.5	Right
	STR-008	37	Female	Hemorrhagic		1	Left

*Note*: AIS = American Spinal Cord Injury Association Impairment Scale; NLI = neurologic level of injury; SCI = spinal cord injury.

### Study design

**Figure 1.**
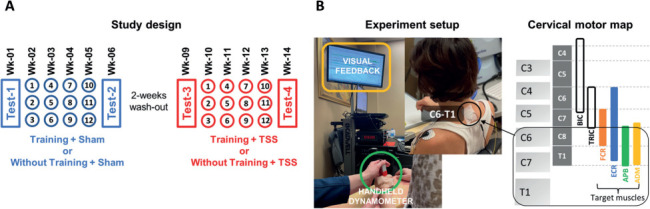
Study design and experimental setup. (**A**) Study design included 12 training sessions incorporating grip training performed in the presence of sham or actual transcutaneous electrical spinal stimulation (TSS) interventions performed as well as four tests and 2 weeks of washout period. The circles in the figure represent the training sessions and are labeled with numbers. (**B**) The experimental setup involved the use of a handgrip dynamometer and visual feedback from electromyography (EMG) activities and grip force signals during training. The cathode electrode was placed between the C6 and T1 vertebrae to target forearm and hand muscles based on the motor pools distributions.

Participants underwent two 4-week interventions consisting of sessions performed three times per week, which included repetitive proximal and distal task-specific movements with or without subsequent hand grip training. The interventions also included either sham TSS (e.g., weeks 2 to 5) or actual TSS (e.g., weeks 10 to 13), counterbalanced across the participants. In this subject-blinded, cross-over design study, the phases were interchanged with a 2-week period of washout/rest between the phases (**Figure 1A**). Based on the previous findings from Inanici et al.,^5^ this washout period is sufficient to distinguish the effects of training with and without electrical neuromodulation. Grip testing occurred in weeks 1, 6, 9, and 14, where hand grip force was evaluated with and without TSS.

### Transcutaneous spinal stimulation

**Figure 3.**
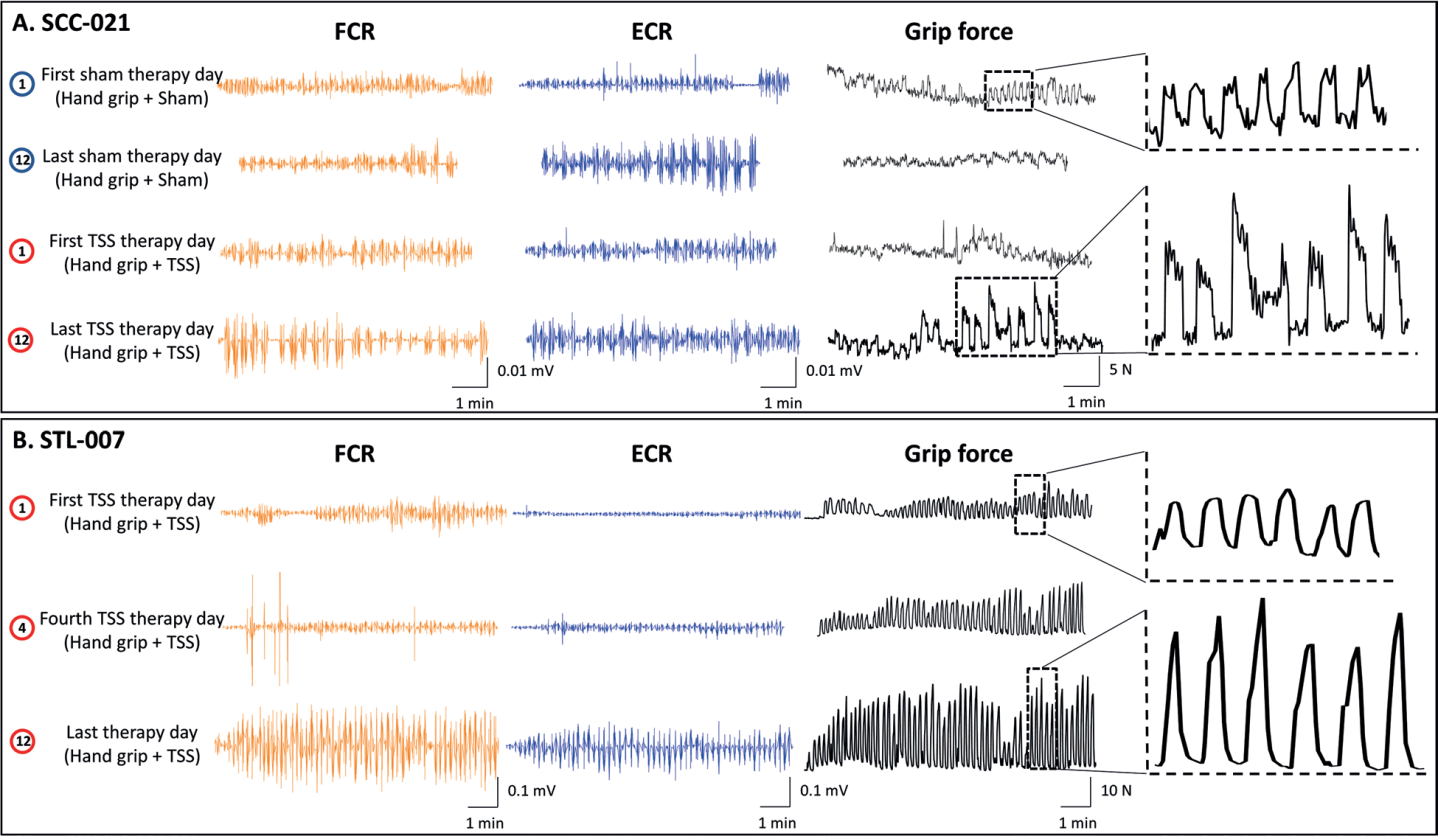
Changes in forearm muscle electromyography (EMG) activities and grip strength during task-specific hand grip training. (**A**) Representative participant in spinal cord injury, SCC-021, during hand grip training within the session of sham transcutaneous electrical spinal stimulation (TSS) and actual TSS. (**B**) Representative participant in stroke, STL-007, during hand grip training within the session of actual TSS. During actual TSS intervention, stimulation waveforms consisted of biphasic 0.5 ms pulses, at a frequency of 30 Hz, delivered over cervical spinal cord between the C6 and T1 spinous processes.

TSS was delivered using a constant-current stimulator DS8R (Digitimer Ltd, UK) via self-adhesive electrodes. A cathode electrode with a diameter of 3.2 cm (PALS, Axelgaard Manufacturing Co. Ltd., USA) was placed on the skin over the cervical spinal cord at midline between the C6 and T1 spinous processes. Two 7.5 × 13 cm self-adhesive oval anodes (PALS, Axelgaard Manufacturing Co. Ltd., USA) were placed on the anterior iliac crests of each participant. The stimulation waveform consisted of biphasic 0.5 ms pulses, and these were delivered at a frequency of 30 Hz with stimulation intensity that was gradually increased and adjusted to enable maximal performance during hand grip test (see **Figure 3A-B**) without causing discomfort (with a range between 20 and 70 mA). During sham TSS, the electrodes were located in the same position, and the intensity of stimulation was set as the same intensity used during TSS intervention; the intensity gradually reduced to zero over a 30-second period.^14^

### Electromyography

Trigno Avanti wireless surface electromyography (EMG) electrodes (Delsys Inc., USA) were placed on the trained UL at four sites: flexor carpi radialis (FCR), extensor carpi radialis (ECR), first dorsal interosseous (FDI), and abductor pollicis brevis (APB) muscles. EMG data was amplified using a Trigno Avanti amplifier (Delsys Inc. USA) and recorded at a sampling frequency of 2000 Hz using a PowerLab data acquisition system (ADInstruments, Australia).

### Grip strength training

The grip strength outcomes in the current study comprised two components: training and testing. During each training session, participants sat in a comfortable upright position with their back straight. They were instructed to hold the handgrip dynamometer (Model: MLT004/ST, ADInstruments, Australia) in the hand being trained. The handgrip dynamometer was adjusted so that the participant was able to grip it comfortably. To ensure accurate measurement and fully enclose the dynamometer, the participants’ hands were affixed to the dynamometer using elastic tape. The participants were instructed to keep their elbow flexed between 90 and 120 degrees and to rest their arms in a comfortable position with a foam pad placed beneath the arms. To minimize compensatory movements involving nontargeted muscles, an additional foam pad was positioned beneath the participants’ wrist and the dynamometer was kept suspended. The participants were then asked to squeeze the dynamometer as quickly and forcefully as possible for 5 to 10 seconds before releasing it. Verbal encouragement was provided to motivate the participants to exert maximal force. In addition, signals from a handgrip dynamometer along with the EMG were recorded and displayed as visual feedback during the training to assist with targeting specific muscles (**Figure 1B**). Participants were provided with EMG activities for four UL muscles: FCR, ECR, FDI, and APB. They also observed a grip force signal accompanied by targeted horizontal lines. These lines were adjusted according to the grip force exerted by the participants, aiming to assist them in achieving their maximum performance.

The grip training began with three to five grip movements without stimulation, followed by at least 20 repetitions of grip movements in the presence of sham or actual TSS intervention. The duration of stimulation during each training session was approximately 10 minutes. During the testing days, participants were asked to perform a total of 9 to 11 hand grip maximal voluntary contraction (MVC) repetitions with and without stimulation to monitor the improvement in their hand grip strength.

### Data processing and analysis

Data were processed using LabChart Pro 8.1.13 software (ADInstruments, Australia). Grip force was measured using a handgrip dynamometer during MVC trials without stimulation and trials with stimulation. The average peak-to-peak amplitude was calculated. Assumptions of normality were tested using the Shapiro-Wilk test. Normally distributed data were analyzed using the repeated measures two-way analysis of variance (ANOVA) to evaluate grip force differences across the testing sessions. Data were corrected for multiple comparisons within figures using a Holm-Bonferroni’s post hoc test. Non-normally distributed data were analyzed with a Kruskal–Wallis test followed by Dunn’s multiple comparison test. *P* values < .05 were considered significant (*p < .05, **p < .01, and ***p < .001). All statistical analyses were performed using OriginPro 2023 (Origin Lab Corporation, Northampton, MA, USA).

## Results

**Figure 2.**
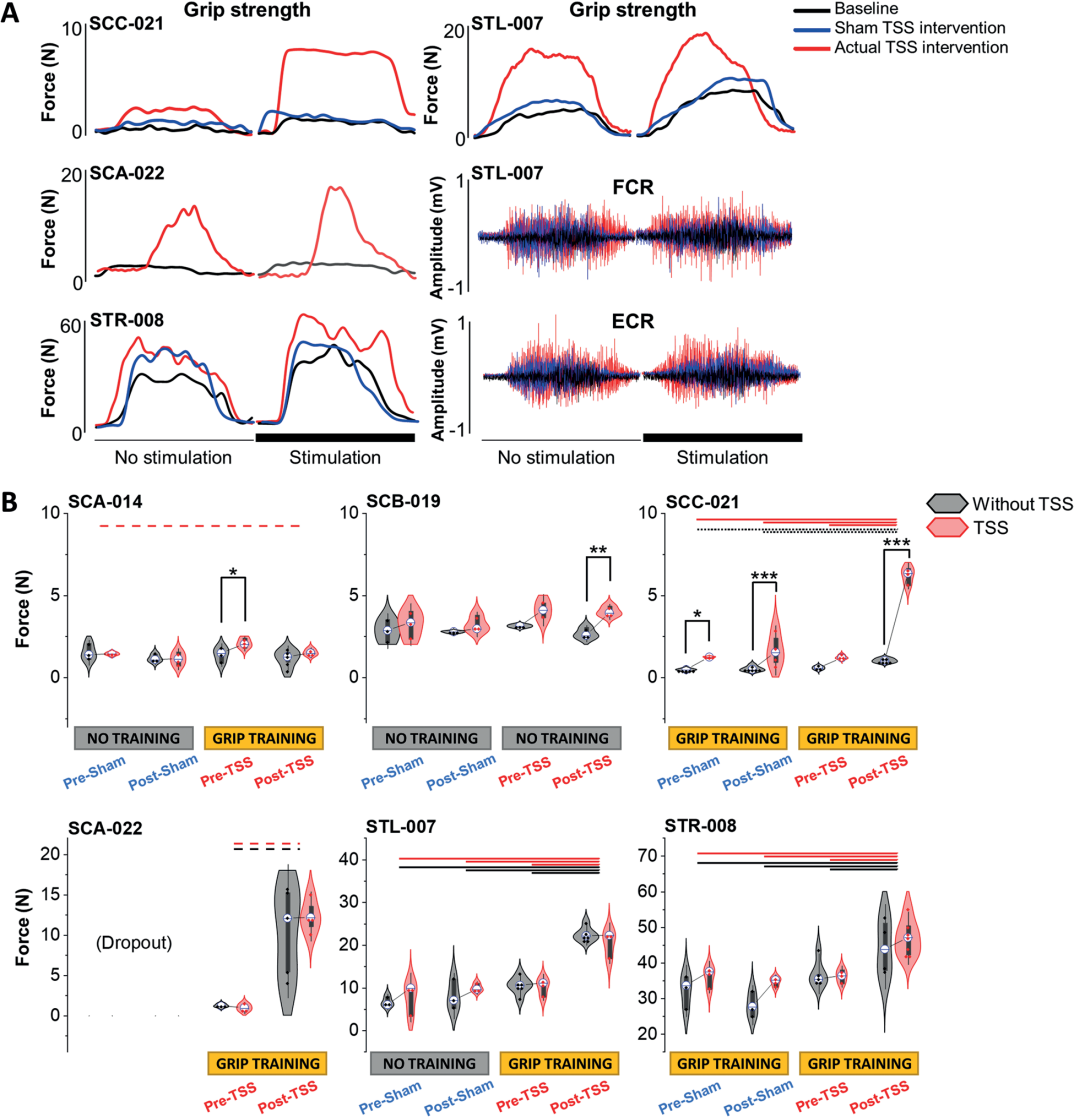
Changes in grip strength in paralyzed individuals. (**A**) Waveforms of grip strength and forearm electromyography (EMG) activities with or without task-specific hand grip training combined with sham transcutaneous electrical spinal stimulation (TSS) and actual TSS intervention in participants after cervical spinal cord injury and stroke who showed improvement across the testing sessions. (**B**) Changes in hand grip strength with or without task-specific hand grip training combined with sham TSS and actual TSS in four participants with cervical SCI classified as AIS A and B and two participants with cerebral stroke. Box range was set as percentage 25% to 75%, white circle in boxplots present the median values, and whiskers indicate the 95% confidence interval. Significant differences across the sessions are indicated with horizontal lines. Dotted lines: *p* < .05; dashed lines: *p* < .01; solid lines: *p* < .001.

At the baseline, only one out of six participants exhibited an increase in grip strength in the presence of TSS compared to without TSS. For participants with SCI, two out of three who underwent the GripTr+TSS intervention demonstrated an increase in the averaged maximum grip force both with and without TSS (**Figure 2A** and **2B**). Specifically, two individuals with cervical SCI (SCC-021 and SCA-022) presented with minimal hand grip strength at baseline. However, following the GripTr+TSS intervention, both participants exhibited significant improvements in their hand grip strength (*p* < .05). Similarly, two participants with stroke experienced similar positive improvements exceeding their baseline performance in their maximum grip force when the GripTr+TSS intervention was employed (*p* < .001). However, sham TSS did not result in any improvement in grip force, regardless of the grip training performed. Notably, participant SCB-019 did not undergo hand grip training for either the sham TSS or actual TSS interventions, resulting in the absence of hand grip force after the NoTr+TSS intervention. Participant SCC-021 demonstrated acute TSS effects on specific testing days during the baseline, post-sham, and post-TSS assessments. However, GripTr+Sham did not lead to any improvement in grip force.

**Figure 3** illustrates the progressive increase in grip force across multiple training sessions during both GripTr+Sham and GripTr+TSS interventions in a representative participant with SCI and during the GripTr+TSS intervention in a representative participant with stroke. Notably, the participant with SCI did not show a significant improvement in grip force on the final day of GripTr+Sham intervention compared to baseline. However, GripTr+TSS intervention resulted in a significant improvement in grip force as well as FCR and ECR muscle EMG activity, surpassing both the baseline and the first day of GripTr+TSS intervention. Similarly, the participant with stroke exhibited a cumulative increase in grip force throughout the GripTr+TSS intervention sessions. On the final day of GripTr+TSS intervention, significant improvements in grip force as well as FCR and ECR muscle EMG activity were observed compared to the first day of intervention.

## Discussion

This study presents preliminary evidence from individuals with SCI and stroke that continuous TSS delivered over the cervical spinal cord, in combination with task-specific hand grip training, resulted in notable improvements in maximum hand grip strength in the trained UL. The results of our case series showed that the individual interventions, either TSS alone in one participant or hand grip training alone in two participants, exhibited limited effectiveness in improving grip strength. Furthermore, to our knowledge, this is the first demonstration of TSS modulating the cervical spinal networks to specifically improve impaired grip strength after stroke.

Recent studies on therapeutic approaches for SCI found that TSS led to an improvement in hand control, hand grip force production, and dexterity measures among individuals with SCI.^5^,^6^,^15^ While many studies have reported improved grip strength as a common outcome, it is still unclear whether grip strength was specifically trained or if it improved in response to spinal neuromodulation alone. Another question that remains from previous research is whether training grip strength alone is sufficient to enhance motor function,^16^ or if combining task-specific training with TSS is necessary to improve motor function.^6^ In the present study, one SCI participant who did not receive hand grip training but did receive TSS demonstrated limited effectiveness of TSS alone in improving hand grip strength. TSS combined with task-specific hand grip training may involve different and perhaps synergistic processes leading to more effective reorganization of neural circuits. In addition, one participant with SCI demonstrated immediate improvements in maximum grip force in the presence of TSS, supporting the concept that TSS can modulate the excitability of neural pathways, which allows for increased access of supraspinal control to cervical sensory-motor networks.^17^ Interestingly, at the last testing day, four participants showed an increase in hand grip MVC even without stimulation with GripTr+TSS intervention, surpassing their baseline performance.

 The extent of enhancement in grip strength varied among individuals with SCI based on the extent and location of the injury^18^ and on the specific neural pathways that were been damaged.^19^ If the injury is too severe or in an area that cannot be effectively stimulated, SCI individuals may not experience significant improvement. For example, participant SCA-014, with the most severe cervical SCI and longest postinjury duration in our group, showed the least improvement compared to other participants. In addition, two participants with stroke showed similar positive improvements in maximum grip force with the TSS intervention. The effectiveness of TSS in two participants with stroke can presumably be attributed to the preservation of spinal circuits.^20^

Limitations of the study include a relatively small sample size, which compromises statistical power and subsequent interpretation of the results. Prior to the grip assessment in the study, the other study protocols that were implemented may have contributed to fatigue, which could have hindered the ability of participants to generate their maximal grip force. We did not apply different stimulation parameters in this study, but instead we focused on demonstrating the efficacy of TSS combined with task-specific training.

## Conclusion

Our findings suggest that a task-specific training protocol involving the combination of TSS and task-specific UL rehabilitation (in this case, hand grip training) could potentially result in significant improvements and facilitate the restoration of motor function in paralyzed limbs in individuals with cervical SCI and cerebral stroke. Further research is needed to determine the optimal stimulation parameters to target specific motor pools and to engage movements in specific UL joints of interest throughout the training process.

## References

[1] Douglas M, Ochoa C, McShan E, Callender L, Froehlich-Grobe K, Driver S (2022). Health-related self-efficacy following stroke, traumatic brain injury, and spinal cord injury. Arch Phys Med Rehabil..

[2] Tsao CW, Aday AW, Almarzooq ZI (2023). Heart disease and stroke statistics—2023 update: A report from the American Heart Association. Circulation.

[3] Barra B, Conti S, Perich MG (2022). Epidural electrical stimulation of the cervical dorsal roots restores voluntary upper limb control in paralyzed monkeys. Nat Neurosci..

[4] Nishimura Y, Perlmutter SI, Fetz EE (2013). Restoration of upper limb movement via artificial corticospinal and musculospinal connections in a monkey with spinal cord injury. Front. Neural Circuits..

[5] Inanici F, Samejima S, Gad P, Edgerton VR, Hofstetter CP, Moritz CT (2018). Transcutaneous electrical spinal stimulation promotes long-term recovery of upper extremity function in chronic tetraplegia. IEEE Trans Neural Syst Rehabil Eng..

[6] Zhang F, Momeni K, Ramanujam A (2020). Cervical spinal cord transcutaneous stimulation improves upper extremity and hand function in people with complete tetraplegia: a case study. IEEE Trans Neural Syst Rehabil Eng..

[7] Kumru H, Rodríguez-Cañón M, Edgerton VR (2021). Transcutaneous electrical neuromodulation of the cervical spinal cord depends both on the stimulation intensity and the degree of voluntary activity for training. A pilot study. J Clin Med..

[8] Harkema S, Gerasimenko Y, Hodes J (2011). Effect of epidural stimulation of the lumbosacral spinal cord on voluntary movement, standing, and assisted stepping after motor complete paraplegia: A case study. Lancet..

[9] Gill ML, Grahn PJ, Calvert JS (2018). Neuromodulation of lumbosacral spinal networks enables independent stepping after complete paraplegia. Nat Med..

[10] Rowald A, Komi S, Demesmaeker R (2022). Activity-dependent spinal cord neuromodulation rapidly restores trunk and leg motor functions after complete paralysis. Nat Med..

[11] Powell MP, Verma N, Sorensen E (2023). Epidural stimulation of the cervical spinal cord for post-stroke upper-limb paresis. Nat Med..

[12] Kumru H, Flores Á, Rodríguez-Cañón M (2021). Cervical electrical neuromodulation effectively enhances hand motor output in healthy subjects by engaging a use-dependent intervention. J Clin Med..

[13] Lu DC, Edgerton VR, Modaber M (2016). Engaging cervical spinal cord networks to reenable volitional control of hand function in tetraplegic patients. Neurorehabil Neural Repair..

[14] Benavides FD, Jo HJ, Lundell H, Edgerton VR, Gerasimenko Y, Perez MA (2020). Cortical and subcortical effects of transcutaneous spinal cord stimulation in humans with tetraplegia. J Neurosci..

[15] Freyvert Y, Yong NA, Morikawa E (2018). Engaging cervical spinal circuitry with non-invasive spinal stimulation and buspirone to restore hand function in chronic motor complete patients. Sci Rep..

[16] Hoffman H, Sierro T, Niu T (2017). Rehabilitation of hand function after spinal cord injury using a novel handgrip device: A pilot study. J Neural Eng..

[17] Hofstoetter US, Freundl B, Binder H, Minassian K (2018). Common neural structures activated by epidural and transcutaneous lumbar spinal cord stimulation: Elicitation of posterior root-muscle reflexes. PLoS One..

[18] Dimitrijevic M, Dimitrijevic M, Illis L, Nakajima K, Sharkey P, Sherwood A (1986). Spinal cord stimulation for the control of spasticity in patients with chronic spinal cord injury: I. Clinical observations. Cent Nerv Syst Trauma..

[19] Nardone R, Höller Y, Taylor A (2015). Noninvasive spinal cord stimulation: Technical aspects and therapeutic applications. Neuromodulation..

[20] Pirondini E, Carranza E, Balaguer J-M (2022). Poststroke arm and hand paresis: Should we target the cervical spinal cord?. Trends Neurosci..

