# Supplementary material for: Combinatorial Effects of Transcutaneous Spinal Stimulation and Task-Specific Training to Enhance Hand Motor Output after Paralysis
Source: Top Spinal Cord Inj Rehabil. 2023 Nov 17;29(Suppl):15–22. doi: 10.46292/sci23-00040S (PMC10759855; doi:10.46292/sci23-00040S)
Supplement: Supplementary file 1 [file i1945-5763-29-suppl-15_s01.pdf]

eTable 1

eTable 1. International Standards for Neurological Classification of Spinal Cord Injury (ISNCSCI) upper limb motor scores for participants with cervical spinal cord injury and Fugl-Meyer Assessment of Upper Extremity Function (FMA-UE) Motor scores for participants with stroke across the sessions

	**ID**	**Pre-Sham**		**Post-Sham**		**Pre-TSS**		**Post-TSS**	
		Left	Right	Left	Right	Left	Right	Left	Right
**SCI** ISNCSCI UL motor	SCA-014	6	5	7	5	7	4	10	5
	SCB-019	13	13	14	14	13	13	13	14
	SCC-021	16	13	15	12	15	13	15	12
	SCA-022[a]	-	-	-	-	12	12	12	9
**Stroke** FMA-UE motor scores	**ID**	**Pre-Sham**		**Post-Sham**		**Pre-TSS**		**Post-TSS**	
	STL-007	30		25		24		31	
	STR-008	27		24		29		30	

Note: ISNCSCI: International Standards for Neurological Classification of Spinal Cord Injury; TSS = transcutaneous electrical spinal stimulation; UL = upper limb.

[a]SCA-022 was not able to complete the phase involving sham TSS due to personal reasons.

This supplementary digital material accompanies
Top Spinal Cord Inj Rehabil 2023;29(suppl):15-22
© 2023 American Spinal Injury Association
www.asia-spinalinjury.org
doi: 10.46292/sci23-00040S
